# Supplementary material for: Evolution recovers the fitness of Acinetobacter baylyi strains with large deletions through mutations in deletion-specific targets and global post-transcriptional regulators
Source: PLoS Genet. 2024 Sep 16;20(9):e1011306. doi: 10.1371/journal.pgen.1011306 (PMC11426457; doi:10.1371/journal.pgen.1011306)
Supplement: Attachment — Submitted filename: Evolved ADP1 Deletions Response 2024-08-31.docx [file pgen.1011306.s007.docx]

We thank the editors and reviewers for their thoughtful questions and suggestions.

Our point-by-point responses are below in blue text.

**Reviewer #1**: This is an interesting and important paper. It reads very well. Genome reduction is an important process in bacterial function and evolution, particularly in the areas of endosymbiosis (where small genomes are an outcome of the process) and synthetic biology (where small genomes are the goal of the process). Understanding how cells adapt to large deletions, whether they be in nature or in the lab, is therefore important and of interest to a broad readership. I do not know of a paper that addresses this issue in an comprehensive manner as this paper. The finding that regulator non-functionalization is the primary means that cells use to compensate for large deletions is very cool.

I only have two small issues.

The first is a lack of citation of a classic related paper. On lines 104-105 in the introduction, the authors state that, “Less is known about whether and when strains with many genes deleted at once can recover wild-type fitness through laboratory evolution.” That’s true, there is not a ton known about this, but I think you are missing perhaps the most relevant example from the literature, Nilsson et al., 2005, Bacterial genome size reduction by experimental evolution. PNAS 102:[12112-12116](http://c05y1x9s.r.us-east-2.awstrack.me/L0/tel:12112-12116/1/010f0191363d4336-452ad522-118d-41f2-bcbc-ef83c9e7cac4-000000/m75R5jg74GpjXsuncZ_9Ib-LkEw=171). Nilsson et al. used a mutator strain of Salmonella to make deletions (in a single-cell bottleneck regime) ranging in size from 1 to 202 kb, and then used experimental evolution (in large population sizes) to show that fitness could be quickly regained in many cases. It’s almost exactly what you see here, although Nilsson and colleagues were not able to sequence the genome, etc., leaving anything about genes and mechanisms of recovery unknown. But still, worth citing and discussing.

**Response**: Thank you for pointing out the omission of the Nilsson *et al.* paper. We had forgotten that they evolved a few of their strains and examined to what extent they regained fitness. We added a mention of this study to the Introduction.

The second is related to lines 415-416, “Three of the most commonly mutated genes (rnd, csrA, and hfq) are all global post-transcriptional regulators of gene expression [32–34].” I think that is true for csrA and hfq, but I am not convinced that this statement is true for rnd. The function of RNase D is a bit elusive, and has been for a long time, but it is clear that it is a 3’ exoribonuclease. My understanding is that it is quite active on tRNA ends, and seems to be involved in 3’ tRNA maturation (although there are a suite of ribonucleases that have overlapping functions in tRNA processing). I agree that some papers, including citation 34 in this paper, suggest that it might be involved in processing other small RNAs in the cell (it most certainly does, most ribonucleases are not finely specific for one molecule). But processing a sRNA in Myxobacteria, a sRNA that is involved in development in that bacterium, does not mean that RNase D is in general a regulator of gene expression, in my view. I do think there is something quite interesting here in the data the authors are seeing related to rnd, but perhaps the strong signal you are seeing is related to tRNA processing or some other secondary global effect that looks like regulation (tRNA processing gets messed up, that changes what the cell is doing, you see a growth effect), but is not itself related to directly binding to transcripts to regulate transcription or translation.

**Response**: The point is well taken that the canonical function of RNase D is in tRNA processing and that this may be driving evolution in our system rather than our hypothesized effects on small RNAs (of which we have no direct evidence, at present). We modified the related portion of the Discussion so that we no longer call RNase D a global regulator and to reflect the additional possibilities related to these mutations affecting its tRNA processing function.

**Reviewer #2**: The manuscript "Evolution recovers the fitness of Acinetobacter baylyi strains with large deletions through mutations in deletion-specific targets and global post-transcriptional regulators" is well-written and includes interesting findings. Here, are my general and specific comments/questions:

- Introduction and Discussion are very long, please condense.

**Response**: We looked at three recent papers in our field published in *PLoS Genetics* (see below). They had Introduction sections with 4-6 paragraphs and 581-1011 words and Discussion sections with 8-12 paragraphs and 1899-2311 words. Our Introduction is 4 paragraphs and 847 words. Our Discussion is 9 paragraphs and 1332 words. On this basis, our Introduction and Discussion sections don’t seem to be unusually long relative to the standards of the journal and our field. Since other reviewers remarked that these sections were “comprehensive”, “well-crafted”, and “illuminating”, we have not edited to reduce their length.

1. Zion, S., Katz, S. & Hershberg, *R. Escherichia coli* adaptation under prolonged resource exhaustion is characterized by extreme parallelism and frequent historical contingency. *PLOS Genetics* 20, e1011333 (2024).

**Introduction**: 6 paragraphs and 1011 words. **Discussion**: 9 paragraphs and 2311 words.

2. Maddamsetti, R. & Grant, N. A. Discovery of positive and purifying selection in metagenomic time series of hypermutator microbial populations. *PLoS Genetics* 18, e1010324 (2022).

**Introduction**: 5 paragraphs and 581 words. **Discussion**: 12 paragraphs and 1899 words.

3. Touchon, M., Perrin, A., de Sousa, J. A. M., Vangchhia, B., Burn, S., O’Brien, C. L., Denamur, E., Gordon, D. & Rocha, E. P. Phylogenetic background and habitat drive the genetic diversification of Escherichia coli. *PLoS Genet* 16, e1008866 (2020)

**Introduction**: 4 paragraphs and 624 words. **Discussion**: 8 paragraphs and 2130 words.

- this might be beyond the scope of this work, but can you test if the gained fitness (evolved strains) is stable or reversible?

**Response:** The evolved mutations, and thus the fitness gains, would be stable if the strains are kept in the same environments in which they evolved. Some of the mutations (e.g., repeat copy number changes and duplications) may be “reversible” in the sense that they could rapidly revert if they were put in another environment in which they were not beneficial or deleterious.

If the question is whether there are necessarily trade-offs such that evolving to recover fitness in one environment would always cause a loss of fitness in other environments (an important question if one wants to make a minimal genome strain that maintains ancestral fitness through a combination of evolution and engineering), that could be tested, but it would require additional experiments on the scale of those conducted in this study. Other bacterial evolution experiments that have switched between multiple environments have generally found that while some beneficial mutations exhibit strong genotype-by-environment interactions, leading to a mutation being beneficial in one environment and deleterious in another one, there are typically enough mutations that do not exhibit these trade-offs that evolution can improve fitness across a range of environments. We do not know if that is the case for the mutations that evolved in our study.

Lines 233-235: "The engineered deletion in MGD12 overlapped the promoter region of murB, which encodes an enzyme involved in peptidoglycan biosynthesis that is essential in A. baylyi ADP1". Does this deletion fuse the murB into another reading frame (that is to say as a result of frame-fusion it might use a secondary promoter)?

**Response:** The MGD12 deletion does not fuse the *murB* gene to an existing reading frame. It places the *murB* gene 244 base pairs downstream of the end of the *dnaX* gene (*ACIAD1970*). The *dnaX* gene has a putative rho-independent terminator eighteen base pairs downstream of its stop codon. It is possible there is enough *murB* expression to support growth of the deletion mutant ancestor (before any compensatory mutations evolve) due to transcriptional readthrough of this *dnaX* terminator and/or the presence of one or more uncharacterized promoters in the portion of the intergenic region upstream of *murB* that remains after this deletion.

- Can you please more clearly explain difference in the evolution in MS versus LB and why? This is in the manuscript but I think it needs to be stated more clearly for readers.

**Response:** Mutations in *rnd* dominated in MS whereas mutations affected a variety of pathways in LB. We modified the Discussion to directly state this difference for readers. We do not, at present, have an explanation for why there were these different evolutionary responses.

- line 307: Did you check the reads to ensure this is not due to the sequencing/assembly errors?

**Response**: This comment refers to this statement: “One of these was a single-base deletion that would result in a frameshift in *znuC*”. Yes, we checked the alignment of reads to the reference genome that was used to make this prediction by examining the HTML output from *breseq.* Furthermore, we checked all of our mutation predictions in each sequenced clone by generating a simulated genome sequence file updated to include the sequence changes caused by the predicted mutations and compared the reads to this sequence in a second round of *breseq* runs to verify that there were no discrepancies. In our experience, this second validation step catches rare cases of alignment errors leading to incomplete/incorrect mutation predictions.

- under "Whole genome resequencing" (Line 666): Please include your reads' QC steps, what programs/settings did you use?

**Response:** As described in the Methods in the following “Mutation analysis” section, Illumina adaptors were trimmed from reads with Trimmomatic. No additional read QC or filtering steps were necessary, which is what we consistently find when analyzing Illumina data with *breseq*. We added the exact options used for Trimmomatic processing to the revised Methods.

**Reviewer #3**: The paper explores the potential for laboratory evolution to restore fitness in Acinetobacter baylyi ADP1-ISx strains with extensive genome deletions. By evolving these strains over roughly 300 generations in both minimal and complex media, researchers observed fitness improvements in most cases. Genome sequencing of the evolved strains revealed mutations associated with different ancestral strains and environments. This study demonstrates how laboratory evolution can restore fitness in strains with reduced genomes, providing valuable insights for synthetic biology applications aimed at developing streamlined microbial chassis.

I find the study design to be well-conceived and the paper clearly written within its chosen structure. The abstract, introduction, and discussion sections are particularly well-crafted and illuminating. The initial results on detected fitness restoration are also very clear.

However, I struggled with the central part of the paper, which discusses the sequencing results. There are pre-existing mutations, mutations that potentially co-occurred in the founding culture, and very few mutations that occurred during the actual evolution. Among these few de novo mutations that evolved in parallel, some are generally beneficial in the corresponding environment. It would be helpful if the paper focused more on the few background-specific mutations that occurred during the evolution experiment. These mutations seem most relevant to the core question of fitness restoration during evolution.

On a related note, considering the paper's focus on fitness restoration after different types of deletions, it seems slightly odd that the strongest adaptive signal highlighted in the abstract pertains to mutations compensating for the restoration of a gene (csrA), which is present in all or most of the different strains tested. This observation about the compensation after restoration of a a gene seem to not directly address the basic question of the paper (mutations beneficial after specific gene deletions).

**Response:** As discussed in the manuscript, the most frequently mutated genes in this experiment (*csrA*, *hfq*, and *rnd*) likely interact in terms of their effects on a common post-transcriptional regulatory network. However, which of these genes mutated (**Figure 4**), as well as the type of mutations in each gene (characterized for *rnd* in **Figure 5** and **Figure 6**)*,* varied across the different deletion backgrounds and environments. Said another way, though mutations in these genes may be generally beneficial, the amount of that benefit varies.

We think it is important to concentrate on what was the dominant response to evolution that we observed, as highlighted in the abstract and summarized in paragraphs 4-6 of the Discussion. To quote from the Discussion, paragraph 6: “Thus, it appears that re-tuning the global regulatory processes (e.g., transcription, translation) is often the most expeditious and generalizable way for evolution to improve the fitness of both wild-type genomes and less-fit reduced genomes.”

We agree that this result—mutations affecting the same genes but differently in the different deletion backgrounds—makes for a more nuanced and complex story than mutations in genes that occur exclusively in a specific deletion background. While we did see several cases of the latter, and we expected them to occur in every deletion background when we designed this experiment, we cannot escape that our results related to fitness recovery were dominated by mutations that were generally beneficial (but to varying extents in different strains/conditions).

The paper provides a detailed description of all mutations significantly associated with different ancestors and environments. At times, this extensive information feels like a long list of anecdotes, making it difficult to parse. I would prefer reducing this part to its essence, perhaps by focusing on the few most important specific mutations and summarizing the rest, relegating detailed information to the supplementary information (SI). Alternatively, mutations could be grouped into fewer classes, each described in detail.

**Response**: We recognize that there are some dense paragraphs describing the pre-existing and significantly associated mutations in the Results. It was quite annoying to us that mutations arose so quickly after constructing the deletions that we had to contend with the complication of the pre-existing mutations (when we would have preferred to only have to do the planned association analysis), but we think it is important that we take time to explain them, since they were clearly important for recovering fitness in certain deletion strains and would be of interest to anyone who wanted to engineer/evolve a minimal *A. baylyi* ADP1 genome in the future.

Since we would have to create a new supplemental text document to make this change, and *PLoS Genetics* does not have strict length restrictions, we prefer to keep the detailed descriptions of these mutations in the main text where readers can skip over them if they wish.

Key to the paper are the association analyses, mostly relegated to the SI, which is common. However, given their importance, I would like to see a more detailed discussion of the statistical power to detect meaningful associations and how this informed the experiment's design. For instance, how does the power to detect associations depend on the number of replicates per ancestor? Was this used to decide on six replicates? Why was only one clone sampled from the endpoint? (I would have expected at least two endpoints to ensure variation is captured both between endpoints and within replicates.) What factors influence the statistical power of the analysis? Can you rule out that some associations of non-preexisting mutations arose from sharing a founding culture (e.g., via Luria-Delbrück effects, which can generate jackpot events)?

**Response**: We evolved as many replicate populations as was experimentally feasible, which was sufficient to find significant statistical associations (**Figure 4**). Other studies (for example, Shoemaker & Lennon 2022 and Deatherage *et al.* 2017, both cited in the manuscript) have already examined some statistical considerations along the lines of what the reviewer suggests. Further developing this type of statistical framework was not the focus of our study. Practically speaking, we can say that a gene had be mutated in at least four replicates of the same ancestor in the same media condition to achieve statistical significance if it was never observed in other treatments. This limit is similar to what was observed in Deatherage *at al.* 2017, which had a design of comparable size (it had six replicates for each of five treatments). Some genes achieve significance with a different number of mutations, depending on how much they are concentrated in a specific subset of ancestor and/or media treatments.

Only one clone was sequenced from each population to avoid pseudo-replication when counting mutations in different genes for the association analysis. Multiple clones picked from the same population are likely to have some of the exact same mutations in common because they share part of their evolutionary history within that population. Since these shared mutations should only be counted once for the association analysis, one generally gains much less information from sequencing the second, third, etc., clones from the same population.

We can rule out the possible effect of founding cultures in most cases because we saw *different* mutations arise in the independent populations derived from the same ancestor. As shown in **Supplementary Table 4** we saw a range of mutations affecting the genes that were significantly associated with certain ancestors, including distinct base substitutions, deletions, and insertions at different locations within their sequences. We noted mutations for which we could not rule this possibility out (i.e., the exact same mutation was present in multiple evolved isolates from one ancestral deletion strain) in **Figure 4** as “pre-existing in ancestor population” mutations.

Other comments:
 * It would be helpful to explain the rationale for using Acinetobacter baylyi. Is it particularly significant or convenient for experiments?

**Response**: *Acinetobacter baylyi’*s natural competence makes it convenient for genome engineering projects, such as creating the deletion strains used as the ancestors of this experiment. *A. baylyi* is also of potential interest for industrial applications due to some of its metabolic capabilities. We added a brief mention of these points to the Introduction.

Figure 4:
 * If strains are missing a horizontal line, does it mean they lack the corresponding gene?

**Response**: No, this does not mean that the gene is deleted in that ancestor strain. Mutations that evolved in only one strain, such as those in *AbsR28* or *murB,* are displayed only for those strains to save space and reduce clutter. If a mutation line is missing for a given ancestor, then it did not ever have any mutations in this gene. We added an explanation to the figure legend.

* Does this graph show all detected mutations? If not, which ones are shown?

**Response**: This figure shows all mutations affecting a gene or group of genes that was mutated in at least three separate evolved isolates. In regard to the earlier question about statistical power, we chose this cutoff for the figure to match how we only performed the association analysis for genes that met this threshold, because genes that experienced fewer mutations could not possibly achieve statistical significance. All mutations we detected are included in **Supplementary Table 4**. We added explanations of both points to the **Figure 4** legend.
